# The effect of obstructive jaundice on the sensitivity of intravenous anesthetic of remimazolam: study protocol for a controlled multicenter trial

**DOI:** 10.1186/s13063-021-05987-y

**Published:** 2022-01-08

**Authors:** Wen Liu, Bin Yang, Jun-Wei Ji, Hua Yang, Hong-Hao Song, Hai-Bo Qiu, Jin-Chao Song

**Affiliations:** 1 Department of Anesthesiology, Shidong Hospital of Shanghai, University of Shanghai for Science and Technology, Shiguang Rd., No. 999, Shanghai, China; 2 Department of Anesthesiology, Chongqing University Cancer Hospital, Chongqing, China; 3 Department of Anesthesiology, Eastern Hepatobiliary Surgery Hospital, Second Military Medical University, Changhai Rd., No. 225, Shanghai, China

**Keywords:** Pharmacodynamics, Remimazolam, Obstructive jaundice

## Abstract

**Background:**

It is well known that obstructive jaundice could affect the pharmacodynamics of some anesthetics, and the sensitivity of some anesthetics would increase among icteric patients. Remimazolam is a new ultra-short-acting intravenous benzodiazepine sedative/anesthetic, which is a high-selective and affinity ligand for the benzodiazepine site on the GABAA receptor. However, no study has reported the pharmacodynamics of remimazolam in patients with obstructive jaundice. We hypothesize that obstructive jaundice affects the pharmacodynamics of remimazolam, and the sensitivity of remimazolam increases among icteric patients.

**Methods/design:**

The study will be performed as a prospective, controlled, multicenter trial. The study design is a comparison of remimazolam requirements to reach a bispectral index of 50 in patients with obstructive jaundice versus non-jaundiced patients with chronic cholecystitisor intrahepatic bile duct stones. Remimazolam was infused at 6 mg/kg/h until this endpoint was reached.

**Discussion:**

Remimazolam could be suitable for anesthesia of patients with obstructive jaundice, because remimazolam is not biotransformed in the liver. Hyperbilirubinemia has been well-described to have toxic effects on the brain, which causes the increasing of sensitivity to some anesthetics, such as desflurane, isoflurane, and etomidate. Furthermore, remimazolam and etomidate have the same mechanism of action when exerting an anesthetic effect. We aim to demonstrate that obstructive jaundice affects the pharmacodynamics of remimazolam, and the dose of remimazolam when administered to patients with obstructive jaundice should be modified.

**Trial registration:**

Chinese Clinical Trial Registry ChiCTR2100043585. Registered on 23 February 2021

## Background

Obstructive jaundice is defined as the retention of bile components and bile after intrahepatic or extrahepatic bile duct obstruction [1]. Patients with obstructive jaundice are prone to acute renal failure, hypotensive shock, sepsis, and multiple organ dysfunction in the perioperative period [2, 3].

Remimazolam is a new, fast-onset, and ultra-short-acting intravenous sedative/anesthetic [4], which is a high-selective and affinity ligand for the benzodiazepine site on the GABAA receptor [5] and rapidly metabolized by tissue esterases to an inactive metabolite [6, 7]. In recent years, there has been a growing interest in the use of remimazolam in general anesthesia and procedural sedation [4]. It is well known that obstructive jaundice could affect the pharmacodynamics of some anesthetics, and the sensitivity of some anesthetics would increase among icteric patients. We have found that patients with obstructive jaundice are more sensitive to etomidate [8]. In the meanwhile, remimazolam and etomidate have the same mechanism of action when exerting an anesthetic effect, which suggests that patients with obstructive jaundice may also be more sensitive to remimazolam.

## Methods/design

### Study settings

**Fig. 1:**
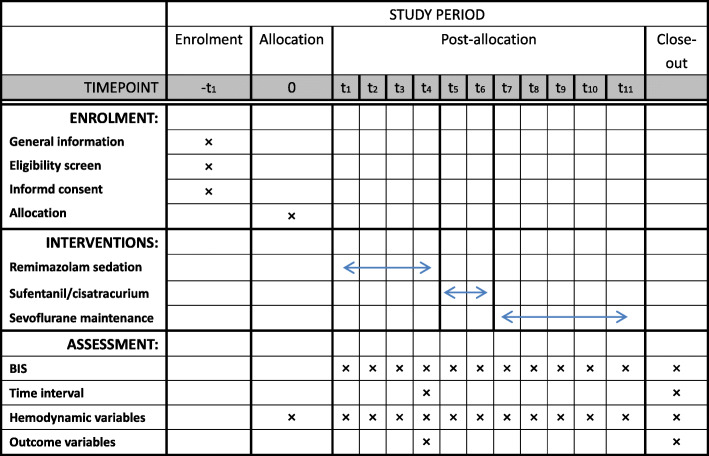
Schedule of enrollment, intervention, and assessment according to the Standard Protocol Items: Recommendations for Interventional Trials (SPIRIT) statement

The study is designed as a prospective, controlled, multicenter trial. It is registered in the Chinese Clinical Trial Registry (ChiCTR2100043585. Date of registration: February 23, 2021). This study mainly aims to investigate whether obstructive jaundice affects the pharmacodynamics of remimazolam and whether the sensitivity of remimazolam increases among icteric patients. It is reported following the SPIRIT reporting guidelines [9]. The sponsor of this trial is the Department of Anesthesiology, Shidong Hospital of Shanghai, University of Shanghai for Science and Technology. The sponsor is responsible for the design, collection, management, analysis, and interpretation of the data; writing; and the decision to submit the report for publication. The study is supported by the Yangpu District Good-Doctor Program funding and The Science and Technology Commission and Health Committee of Yangpu District, Shanghai (YPM202105). Figure 1 shows the SPIRIT checklist that we follow in this report.

### Participants

#### Number of patients needed

We plan to divide the patients into the obstructive jaundice group (total bilirubin (TBL) value > 17.1 μmol/L) and the control group (TBL < 17.1 μmol/L) based on their diagnosis and TBL value. The primary endpoint is the difference in the requirement of remimazolam.

The group sample size was calculated based on the differences in remimazolam requirement to reach BIS value ≤ 50 longer than 5 s in our previous study, in which the mean remimazolam requirement was 0.13 ± 0.04 mg/kg (*n* = 6) in the obstructive jaundice group and 0.16 ± 0.04 mg/kg (*n* = 6) in the non-obstructive jaundice group, The following formula: *n* = 15.7/ES^2^ + 1, where ES = effect size = (difference between the groups)/(mean of the SD between the groups), with *α* = 0.05 and power = 0.8, was used to determine that the study would be adequately powered with *n* = 29 per group. Considering a dropout rate of 10%, the estimated sample size will be at least 32 patients per group; thus, a total of 64 patients will be needed.

#### Eligibility

The study takes place at three centers: the Department of Anesthesiology, Shidong Hospital of Shanghai, University of Shanghai for Science and Technology; Department of Anesthesiology, Eastern Hepatobiliary Surgery Hospital, Second Military Medical University; and Department of Anesthesiology, Chongqing University Cancer Hospital beginning March 2021 to March 2022. Eligible patients for participation in this clinical trial are 32 patients with obstructive jaundice (serum TBL > 17.1 μmol/L) secondary to neoplasm of the bile duct or the head of the pancreas and 32 non-jaundiced patient controls with chronic cholecystitisor intrahepatic bile duct stones. All patients enrolled in this clinical trial are aged 18 to 75 years, American Society of Anesthesiologists (ASA) grades II to III, and given written informed consent, who are scheduled to undertake surgery.

#### Exclusion criteria

Patients are excluded if the following criteria appear in their medical history:

- Age range < 18 years or > 75 years
- ASA physical status I, IV, or V
- Weight beyond ± 20% of ideal
- Allergic reaction to the planned medication
- History of psychological problems or psychiatric disease
- Using any form of analgesic or neuromodulating medications
- Known or suspected cardiac, pulmonary, renal, or metabolic disease

The schedule of enrollment, intervention, and assessment is reported according to the SPIRIT statement (Fig. 1).

The number of excluded patients and the reasons for their exclusion will be reported according to the SPIRIT statement.

#### Consent

Written consent was obtained from all patients. The patient’s history and current health status are screened during the standard anesthesia evaluation before the surgery. The investigators use anesthesia pre-assessment sheets to screen patients for inclusion and exclusion criteria. Inclusion will not be finalized until the patient signs the informed consent on the day of surgery.

If patients refuse to participate in the study, they will be sedated with propofol according to the anesthetic standards. The investigator or physician who examines the subject may decide to remove the subject from the study if the subject has an emergency medical problem (allergic reaction or acute health problem).

### Ethical approval

The trial is approved by the Committee on Ethics of Biomedicine Research, Shidong Hospital Affiliated to University of Shanghai for Science and Technology (YPSDKY2020-004-010).

### Allocation and allocation concealment

Patients are allocated to the obstructive jaundice group or the control group based on the TBL values after they sign the informed consent form. Because obstructive jaundice is easy to distinguish from the appearance of skin color, it is difficult to double-blind. However, the anesthesiologist assistant is blind to the grouping or induction method but only records the data through a local area network (LAN) in the next room.

### Intervention

After 8 h of fasting, the patients without premedication will be brought into a quiet operating room where a cannula was inserted into the right internal jugular vein under local anesthesia for infusion of remimazolam and liquid. Radial artery catheterization was placed to measure invasive arterial blood pressure. Heart rate (HR), invasive blood pressure (IBP), electrocardiogram (ECG), end-tidal carbon dioxide (ETCO_2_), and oxyhemoglobin saturation (SpO_2_) are routinely monitored during the whole process of research (Philips HP Viridia24/26 M1205A). Apply the BIS sensor (BIS™XP sensor) as recommended by the manufacturer. The patients are asked to keep their eyes closed and covered with gauze to avoid any interference with sound and light stimulation. The temperature of the room is controlled at 23 °C.

The study is designed to record the remimazolam requirement with BIS of 50 as the endpoint. The patients will be treated with remimazolam at a rate of 6 mg/kg/h by a Graseby 3500 syringe pump (SIMS Graseby Ltd., Herts, UK) until the BIS is ≤ 50 longer than 5 s. The assistant anesthesiologist, who is not aware of the study group, observes the vital signs and BIS values in the next room via the LAN, determines the endpoint of titration, and records the dosage of remimazolam used and the time interval between the start and end of the infusion. Then, the patients will be given 0.4–0.6 μg/kg sufentanil and 0.2 mg/kg cisatracurium, and endotracheal intubation will be performed 3 min later for anesthesia induction. Anesthesia is maintained with sevoflurane (1.5–2.5%) at an appropriate standard for surgical procedures. Hemodynamic data are collected and recorded at relevant points during the peri-intubation period.

### Primary objective

#### Definition of primary endpoint

The primary endpoint of the study—reflecting the change of sensitivity of remimazolam in patients with obstructive jaundice—is remimazolam requirement with BIS of 50 as the endpoint.

#### Assessment of primary endpoint

We will record the requirement of remimazolam and the time interval between the start and end of the infusion.

### Secondary objectives

#### Definition of secondary endpoints

Secondary endpoints focus on hemodynamic stability and safety, which is reflected in the number of cardiovascular events and the average percent change to baseline in the mean arterial pressure and heart rate.

#### Assessment of secondary endpoint

Hemodynamic data at the designated time points will be recorded during the perintubation period.

### Safety management and adverse event

Researchers make sure that emergency equipment is working throughout the process. An adverse event refers to any untoward medical occurrence which happens during the trial. Adverse events include but are not limited to respiratory depression or cardiocirculatory instability. All adverse events will be treated immediately. If spontaneous ventilation is insufficient (SpO_2_ < 92%), auxiliary mask ventilation is given to the patients when necessary to maintain ETCO_2_ between 34 and 45 mmHg. Cardiovascular events should be handled promptly, with 5–15 mg ephedrine given if the patient’s blood pressure is below 60 mmHg and 5 mg atropine given if the patient’s heart rate is below 50 bpm. The type of adverse event, likely cause, and treatment will be documented and discussed in data monitoring committee (DMC) meetings.

### Data collection and data management

Before surgery, general information and the surgical methods of the patients are investigated, and indexes such as TBL, bile acid, albumin, alanine aminotransferase (ALT), aspartate aminotransferase (AST), and alkaline phosphatase (ALP) are recorded. The requirement of remimazolam until the patient’s BIS value reaches 50 and the time interval from the start to the end of infusion are recorded. Pulmonary and cardiovascular vital signs are recorded electronically throughout the procedure, including SpO_2_ measured by a pulse oximeter, HR, IBP, RR, and ETCO_2_. Data will be collected on paper case report forms (CRFs) by an anesthesiologist assistant blinded to the group allocation in each center.

### Data monitoring

The data monitoring committee, composed of statisticians, representatives from the ethics committee, and principal investigators from each center, will be responsible for data monitoring. The members of the DMC are independent of the sponsors. Written reports on trial progress will be submitted to the committee quarterly. Cases of unexpected scenarios and adverse events will be discussed at committee meetings.

### Statistical analysis

Statistical analyses will be performed by an independent statistician using the SPSS 19.0 statistical software. The measurement data of normal distribution will be presented as mean ± standard deviation. Independent sample *T* test or rank-sum test will be used for comparison between the groups, and multiple linear regression analysis will be used to test the relationship between the dosage of remimazolam and TBL, TBA, ALB, AST, and ALT.

## Discussion

Obstructive jaundice may result in hepatic cell damage and hepatosis through various mechanisms [10]. Alanine aminotransferase, aspartate aminotransferase, and alkaline phosphatase increased in obstructive jaundice patients [8, 11]. The drug-metabolizing enzyme systems may be impaired in patients with obstructive jaundice. Plasma clearance of drugs such as dexmedetomidine and rocuronium, which are metabolized and excreted by the liver, is decreased significantly in patients with obstructive jaundice. Furthermore, cardiovascular abnormalities have been well known to occur in obstructive jaundice patients for many years [12–14]. It was reported that internal biliary drainage produces an improvement in hemodynamics [15].

Remimazolam could be suitable for anesthesia of patients with obstructive jaundice, because remimazolam is not biotransformed in the liver. Schüttler et al. reported that remimazolam has a high clearance (1.15 ± 0.12 L/min), a small steady-state volume of distribution (35.4 ± 4.2 L), and a short terminal half-life (70 ± 10 min). The simulated context-sensitive halftime after an infusion of 4 h was 6.8 ± 2.4 min [16]. It was reported that remimazolam, which did not accumulate after prolonged infusion, could be used for the maintenance of general anesthesia in many nations [4, 17]. Furthermore, remimazolam was characterized by moderate hemodynamic side effects [16]. A multicenter, randomized, double-blind, parallel-group trial demonstrated remimazolam’s safety and efficacy in vulnerable patients (ASA class III) undergoing elective general surgery [18]. All these profiles of remimazolam determine its position in anesthesia for patients with obstructive jaundice, in which patients the duration of the operation is often relatively long and the hemodynamics is relatively unstable.

Hyperbilirubinemia has been well-described to have toxic effects on the brain. Histologic evidence of neurologic damage in the brain, such as atrophy and pyknosis of nerve cells, ghost cells, and neuronophagia, was observed in a well-designed canine model of cholestasis secondary to bile duct resection. Furthermore, neurologic damage is more widespread as the duration of hyperbilirubinemia is prolonged [19]. It has been reported that interaction of unconjugated bilirubin with synaptosomal membrane vesicles results in oxidative injury, calcium intrusion, and loss of membrane asymmetry and functionality, thus potentially contributing to the pathogenesis of encephalopathy by hyperbilirubinemia [20]. Acute hyperbilirubinemia results in presynaptic neurodegeneration at a central glutamatergic synapse [21]. Unconjugated bilirubin could also impair the release and uptake of the neurotransmitter glutamate, indicating possible excitotoxic damage [22, 23]. It has been reported that altered neurotransmission could occur in the brains of cholestatic patients. Some evidence suggests that fatigue and pruritus accompanying with cholestasis may be the consequences of a central mechanism, increased opioidergic neurotransmission [24, 25], and defective serotoninergic neurotransmission [26–28]. Consequently, all these changes in patients with obstructive jaundice may, at least in part, induce the increased sensitivity to anesthetics and the reduction of anesthetic requirements.

Our previous study demonstrates that the MACawake of desflurane was significantly decreased in obstructive jaundice patients compared with non-jaundiced controls; furthermore, there is a highly inverse relation between the MACawake of desflurane and the concentration of serum total bilirubin [29]. We also demonstrated that patients with obstructive jaundice had an increased sensitivity to isoflurane, more bradycardia and hypotension during anesthesia induction and maintenance, and a prolonged recovery time compared with non-jaundiced patients [30].

In our other research, it was shown that the etomidate requirement was significantly decreased in patients with obstructive jaundice, and there was a significant negative correlation between serum total bilirubin and etomidate requirement [8]. Etomidate is a pure hypnotic GABA agonist [31–33]. Shi et al. demonstrated that bilirubin potentiates inhibitory synaptic transmission in the lateral superior olive neurons of rats, and the potentiation of depolarizing GABA/glycinergic transmission by bilirubin could underlie bilirubin excitotoxicity [34, 35].

It just so happens that the remimazolam we are discussing is also a high-affinity and selective ligand for the benzodiazepine site on the GABAA receptor. Moreover, remimazolam does not show selectivity between GABAA receptor subtypes [5]. In other words, remimazolam and etomidate have the same mechanism of action when exerting an anesthetic effect. Patients with obstructive jaundice are more sensitive to etomidate [8], which suggests that patients with obstructive jaundice may also be more sensitive to remimazolam.

In conclusion, the toxic effects of hyperbilirubinemia on the central nervous system, such as histologic evidence of neurologic damage, altered neurotransmission in the brain, presynaptic neurodegeneration at the central glutamatergic synapses, and impairment of the release and uptake of the neurotransmitter glutamate, may induce the increased sensitivity to anesthetics and the reduction of anesthetic requirements.

The aim of our trial is to show that obstructive jaundice affects the pharmacodynamics of remimazolam, and the sensitivity of remimazolam increases among icteric patients; thereby, anesthesiologists caring for patients with obstructive jaundice should be alert to the interaction of bilirubin and remimazolam sensitivity.

### Trial status

The first patient was included on 1 March 2021. We expect to finalize the study in March 2022.

### Conflict of interest statement

There are no commercial or financial interests involved in this work.

## Data Availability

Not applicable.
